# Pulmonary paracoccidioidomycosis in AhR deficient hosts is severe and associated with defective Treg and Th22 responses

**DOI:** 10.1038/s41598-020-68322-6

**Published:** 2020-07-09

**Authors:** Eliseu Frank de Araújo, Nycolas Willian Preite, Marc Veldhoen, Flávio Vieira Loures, Vera Lúcia Garcia Calich

**Affiliations:** 1 Departamento de Imunologia, Instituto de Ciências Biomédicas, Universidade de São Paulo, São Paulo, SP Brazil; 2 Instituto de Medicina Molecular Joâo Lobo Antunes, Faculdade de Medicina da Universidade de Lisboa, Lisbon, Portugal; 3 Instituto de Ciência E Tecnologia, Universidade Federal de São Paulo, São José dos Campos, SP Brazil

**Keywords:** Immunology, Infectious diseases, Fungal infection

## Abstract

AhR is a ligand-activated transcription factor that plays an important role in the innate and adaptive immune responses. In infection models, it has been associated with host responses that promote or inhibit disease progression. In pulmonary paracoccidioidomycosis, a primary fungal infection endemic in Latin America, immune protection is mediated by Th1/Th17 cells and disease severity with predominant Th2/Th9/Treg responses. Because of its important role at epithelial barriers, we evaluate the role of AhR in the outcome of a pulmonary model of paracoccidioidomycosis*.* AhR^−/−^ mice show increased fungal burdens, enhanced tissue pathology and mortality. During the infection, AhR^−/−^ mice have more pulmonary myeloid cells with activated phenotype and reduced numbers expressing indoleamine 2,3 dioxygenase 1. AhR-deficient lungs have altered production of cytokines and reduced numbers of innate lymphoid cells (NK, ILC3 and NCR IL-22). The lungs of AhR^−/−^ mice showed increased presence Th17 cells concomitant with reduced numbers of Th1, Th22 and Foxp3^+^ Treg cells. Furthermore, treatment of infected WT mice with an AhR-specific antagonist (CH223191) reproduced the main findings obtained in AhR^−/−^ mice. Collectively our data demonstrate that in pulmonary paracoccidioidomycosis AhR controls fungal burden and excessive tissue inflammation and is a possible target for antifungal therapy.

## Introduction

AhR is a ligand-activated cytosolic transcription factor with known involvement in the metabolism of xenobiotic compounds. It mediates the toxic effects of man-made aryl hydrocarbons including 2,3,7,8-tetrachlorodibenzeno-p-dioxin (also known as TCDD or Dioxin)^1^ but can also be activated by a diverse set of endogenous and exogenous ligands^2^. In steady state, AhR remains in the cytoplasm as part of an inactive complex composed of several chaperone proteins^3^. Upon ligand binding, AhR is released, translocate to the nucleus and heterodimerizes with its partner AhR Nuclear Translocator (ARNT). The heterodimer can bind genomic regions containing its binding motif, thereby inducing the transcription of target genes including detoxifying enzymes of the cytochrome P_450_ family^4^ and the AhR repressor that disrupts the AhR/ARNT complex and attenuates AhR signaling. In the nucleus, AhR can interact with several transcription factors that regulate its activity^3^. The degree of AhR activation may dependent on the structure and receptor affinity of the ligand. Several tryptophan (Trp) degradation products such as Kynurenine (Kyn), produced by the enzymatic action of indoleamine 2,3 dioxygenase (IDO-1) and 6-formylindolo[3,2-b] carbazole (FICZ), a tryptophan condensation product, are well known AhR ligands^5,6^.

AhR is expressed by cells of the innate and adaptive immune system and participates in the control of cell proliferation, differentiation and cytokine secretion^6–10^. In 2008, different laboratories showed that AhR contributes to the differentiation of Th17 and regulatory T cells (Treg)^8–10^. Interestingly, two different AhR ligands (TCDD and FICZ) were shown to modulate the severity of experimental autoimmune encephalomyelitis in opposing directions^9,10^. These findings led several investigators to study the role of AhR in diverse autoimmune, neoplastic and infectious diseases. However, the effects of AhR activation were not homogeneous due to the diverse AhR ligands used, type of pathology studied, and treatment protocols used^3,11–14^.

Many studies have tested the influence of AhR activity and its ligands in infectious processes^12^. Several reports have demonstrated the regulatory activity of AhR on viral^15^, bacterial^14,16^, protozoal^17^, parasitic^18^ and fungal^19–22^ infections. For example, in *Candida albicans* and *Aspergillus fumigatus* infections, the IDO enzyme was shown to concomitantly reduce fungal loads mediated by Trp starvation and inflammatory reactions due to the expansion of Treg cells via Kyn-induced AhR activation^20–22^. Besides Th17/Treg cell differentiation^9,10^, agonist-activated AhR was shown to be linked with IL-22 production^8,23,24^. Indeed, at mucosal sites infected with *C. albicans* AhR was shown to control IL-22 transcription/production that provides a protective phenotype in intestinal epithelial cells^22^. AhR was also involved in the expansion and differentiation of innate lymphoid cells (ILCs), a family of immune cells that do not express rearranged antigen receptor but can be organized with phenotypes similar to Th subsets^25–29^.

Paracoccidioidomycosis (PCM) is the most prevalent systemic granulomatous mycosis in Latin America affecting immunocompetent individuals^30^. It is caused by the dimorphic fungi of the *Paracoccidioides* genus (*P. brasiliensis* and *P. lutzii*)^31^. The infection is acquired by the respiratory route after inhalation of fungal conidia. In the lungs, the fungal propagules can be controlled by resident innate immune cell, can promote localized lesions, or can disseminate via hematogenous or lymphatic systems.

^30^. *P. brasiliensis* infection results in three well defined outcomes: the subclinical infection, where no symptoms are present but might evolve towards a progressive, clinically manifested disease; the acute form, which generally affects young individuals of both sexes, is an overt and severe process evidenced by involvement of multiple organs; the chronic form, the most common (90%) clinical presentation observed in older individuals, predominantly men, presents heterogeneous clinical manifestations, ranging from isolated pulmonary or epithelial lesions (unifocal form) to systemic involvement (multifocal form) ^32^. Similar to other systemic mycoses, immunoprotection in PCM is mediated by Th1/Th17 responses that are controlled by the anti-inflammatory activity of Treg cells. The severe forms are associated with Th2/Th9 responses and excessive expansion of Treg cells^33^.

Our laboratory developed a murine model of PCM that mimics the main findings of the human disease. The mild disease developed by genetically resistant mice involve the preferential activation of Th1/Th17 lymphocytes, while deficiency in IFN-g production associated with prevalent Treg cell activation leads to severe disease^34,35^. Our recent studies have demonstrated that *P. brasiliensis* infection induces a marked IDO-1 expression that mediates Trp catabolism resulting in increased Kyn production and AhR activation^36^. *P. brasiliensis* uses two distinct mechanisms to trigger IDO-1 expression that is dependent on the genetic pattern of hosts. In susceptible mice (B10.A), IDO-1 is induced by IFN-γ and predominantly exhibits enzymatic activity. In resistant (A/J) mice, activation of dendritic cells mediated by TGF-β results in IDO-1 phosphorylation that confers a signaling property to the molecule and a tolerogenic profile for plasmacytoid dendritic cells^36,37^. Our studies have also demonstrated that IDO-1 and AhR are mutually regulated and influence the Th17/Treg cell balance^38,39^. Indeed, in pulmonary PCM absence of IDO-1 expression leads to a robust reduction of AhR expression that results in uncontrolled fungal growth associated with severe tissue pathology mediated by dysregulated Th17/Treg cell immunity^39^. However, the exact role of AhR in the immunoregulation of pulmonary PCM was not still defined. Using AhR^−/−^ mice, we could demonstrate that AhR expression is important for the control of fungal growth and differentiation of ILCs and T cells. Pulmonary PCM in AhR^−/−^ mice is characterized by progressive fungal growth and dissemination despite enhanced Th17 cell immunity that, instead of immunoprotection, appears to be involved in tissue damage and disease severity. The robust influence of AhR in IL-22 producing cells was also demonstrated: a decreased migration of CD4^+^IL-22^+^ T lymphocytes, ILC3 and NCR^+^ IL-22^+^ ILCs were observed in the lungs AhR^−/−^ mice. Importantly, the main findings with AhR^−/−^ mice were validated with an AhR-specific antagonist, the drug CH223191, indicating that immunomodulation of AhR could be used in future immunotherapeutic procedures.

## Results

### Absence of AhR increases fungal loads and tissue pathology reducing the survival of *P. brasiliensis* infected mice

**Figure 1:**
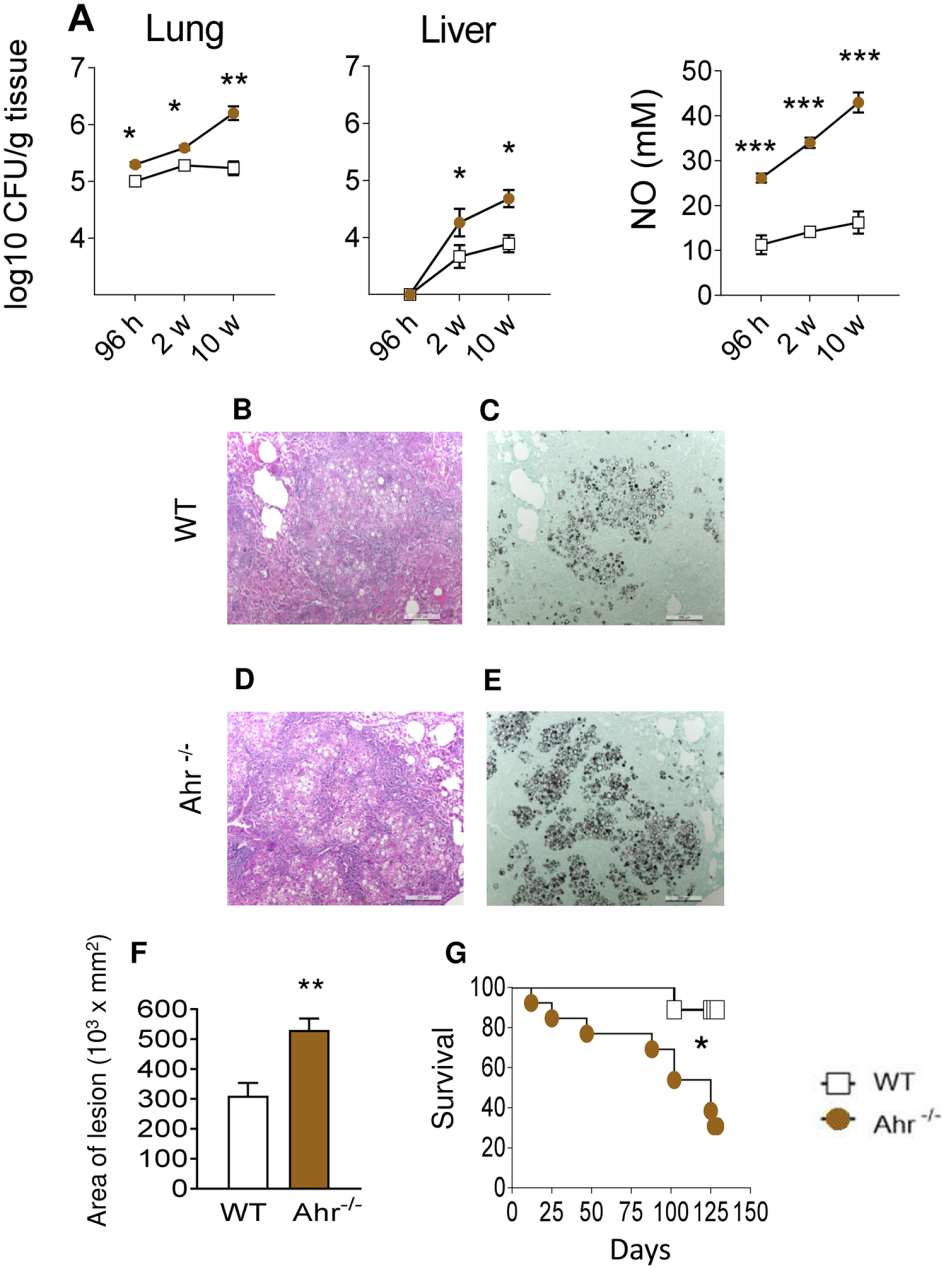
Absence of AhR increases fungal loads, lung lesions and mortality of *P. brasiliensis* infected mice. Groups of 5 AhR^−/−^ and WT C57BL6/J mice were infected with 1 × 10^6^ *P. brasiliensis* yeasts and disease severity was assessed at 96 h, 2 and 10 weeks after infection. (**A**) Fungal load was measured in the lungs and liver of mice via Colony Forming Units (CFU) assay and expressed as log_10_ CFU/g of tissue. The levels of nitric oxide (NO) were quantified in lung supernatants by a standard Griess reaction. (**B**–**E**) Photomicrographs of lung lesions of WT (**B**, **C**) and AhR^−/−^ (**D**, **E**) mice at week 10 after infection. The lung sections were stained with hematoxylin–eosin (left panels) to analyze the presence of inflammation or silver stained by Grocott (right panels) for the presence of fungal cells. (**F**) Morphometric analysis of lung lesions analyzed at week 10 after infection. The lesion areas were measured in mm^2^ in 10 microscopic fields per slide in 5 mice per group and were expressed as mean ± SEM. (**G**) Survival curves of AhR^−/−^ and WT mice were determined in a period of 140 days. Experiments were repeated three times. The asterisk (*) represents statistically significant difference (*P < 0.05; **P < 0.01 and ***P < 0.001).

The influence of AhR on the fungal burden, recovered 96 h, 2 and 10 weeks after infection with 1 × 10^6^ *P. brasiliensis* yeasts, was assessed by a CFU assay. These time points were used to better assess some features of innate (96 h), and early (2 weeks) and late phases (10 weeks) of adaptive immunity. Compared to WT controls, AhR^−/−^ mice showed higher pulmonary and hepatic fungal loads (Fig. 1A). The levels of pulmonary NO after 96 h, 2 and 10 weeks of infection was also higher in AhR^−/−^ mice (Fig. 1A), indicating that fungicidal ability in the absence of AhR is reduced, despite increased levels of nitric oxide that is considered a major *P. brasiliensis* fungicidal mediator of phagocytic cells^40^.

The lung histopathology of control and AhR^−/−^ mice was evaluated after 10 weeks of infection and a higher number of yeast cells was observed in the lungs of the AhR-deficient mice (Fig. 1B–E). These increased fungal loads were associated with larger lesion areas in the lungs (Fig. 1F), which likely contributed to the reduced survival rates of AhR^−/−^ mice (Fig. 1G).

### AhR-deficiency increases the number of pulmonary CD11c^+^ leukocytes with activated phenotype

**Figure 2:**
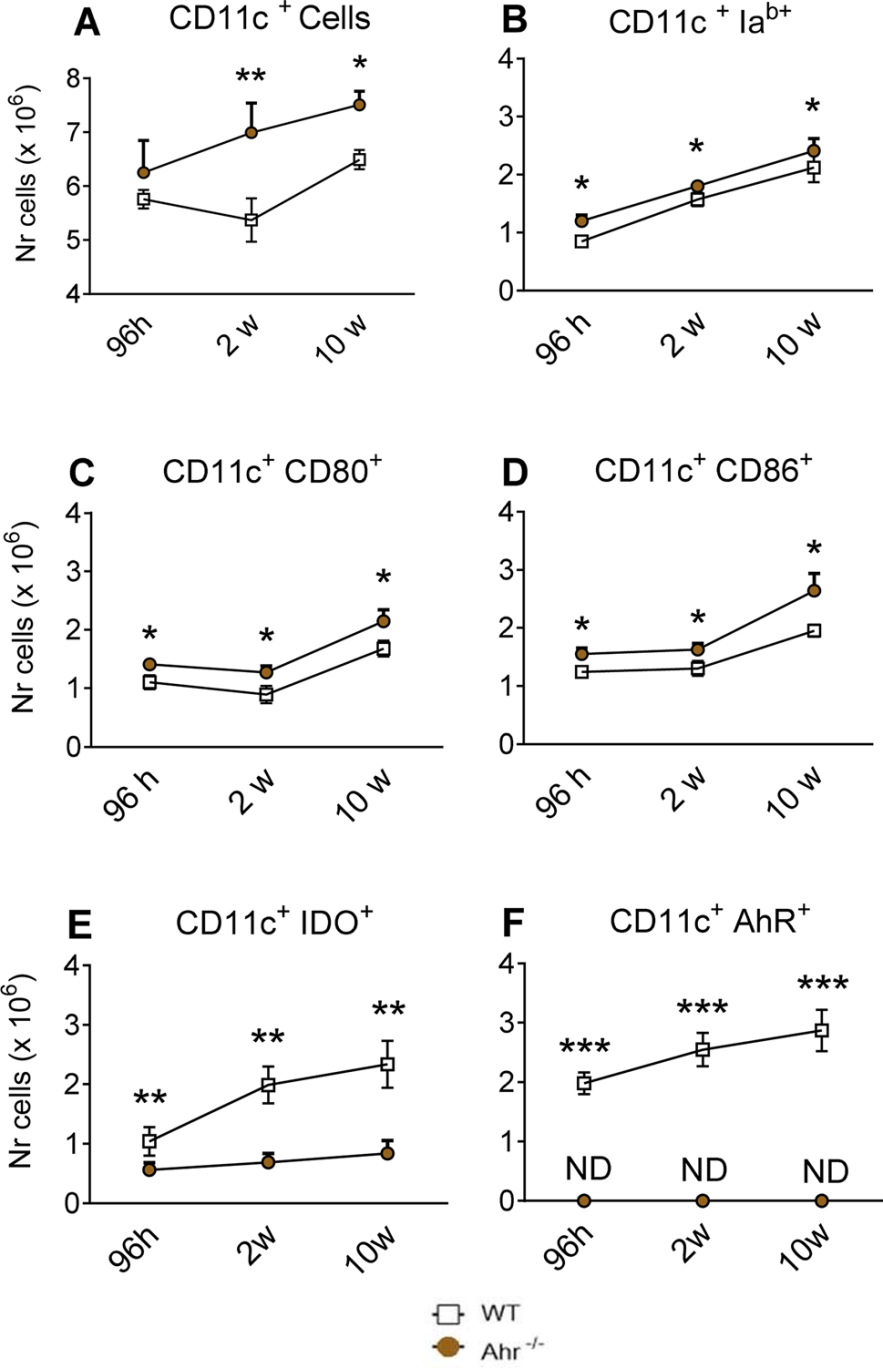
Absence of AhR increases the expression of activation markers (IA^b^, CD86, CD80) but reduces the number of myeloid CD11c^+^ cells expressing indoleamine 2,3 dioxygenase (IDO-1) upon *P. brasiliensis* infection. The number of total CD11c^+^ myeloid cells (**A**) expressing activation markers IA^b^, CD80, CD86 (**B**–**D**), the enzyme IDO-1 (**E**) and the transcription factor AhR (**F**) was characterized by flow cytometry in lung leukocytes from AhR^−/−^ and WT mice obtained 96 h, 2 and 10 weeks after infection with 1 × 10^6^ viable yeasts of *P. brasiliensis*. The lung infiltrating leukocytes were gated by FSC/SSC analysis. The cells were then gated for CD11c expression and the number of cells expressing activation markers and proteins was then assessed. The data represent the mean ± SEM of 3 experiments using 5 mice per group. The asterisk (*) represents a statistically significant difference (*P < 0.05 and **P < 0.01).

C57BL6/J WT and AhR^−/−^ mice were infected as previously described, their lung cells obtained after 96 h, 2 and 10 weeks of infection and analyzed for expression of surface molecules by flow cytometry. CD11c^+^ myeloid cells were chosen because this cell marker is highly expressed in dendritic cells and our previous studies have shown their high involvement in AhR/IDO expression^36,38,39^. Thus, lung CD11c^+^ cells were assessed for the expression of IA^b^, CD86 and CD80 as activation markers^41^. As can be seen in Fig. 2A, AhR^−/−^ mice presented increased influx of CD11c^+^ lung myeloid cells expressing MHC class II molecules (IA^b^) and costimulatory (CD80 and CD86) molecules (Fig. 2B–D) indicating a higher activated phenotype than those of control WT mice.

### Absence of AhR reduces numbers of IDO-1-expressing myeloid cells

Previous reports demonstrating that immunoregulation exerted by IDO-1 on innate and adaptive immunity depends on the activation of the AhR^3,11^, led us to examine the numbers of CD11c^+^ cells expressing IDO-1 and AhR in AhR^−/−^ and WT infected mice. Lung leukocytes were obtained 96 h, 2 and 10 weeks after infection, and the number of IDO-1^+^ and AhR^+^ cells was determined by flow cytometry. Compared with WT controls, a significant reduction of IDO-1^+^CD11c^+^ cells was detected in AhR^−/−^ mice. As expected, only WT mice expressed AhR^+^ cells that increased in the course of infection (Fig. 2E, F).

### Characterization of cytokines in CD11c^+^ lung leukocytes from infected AhR^−/−^ and control mice

**Figure 3:**
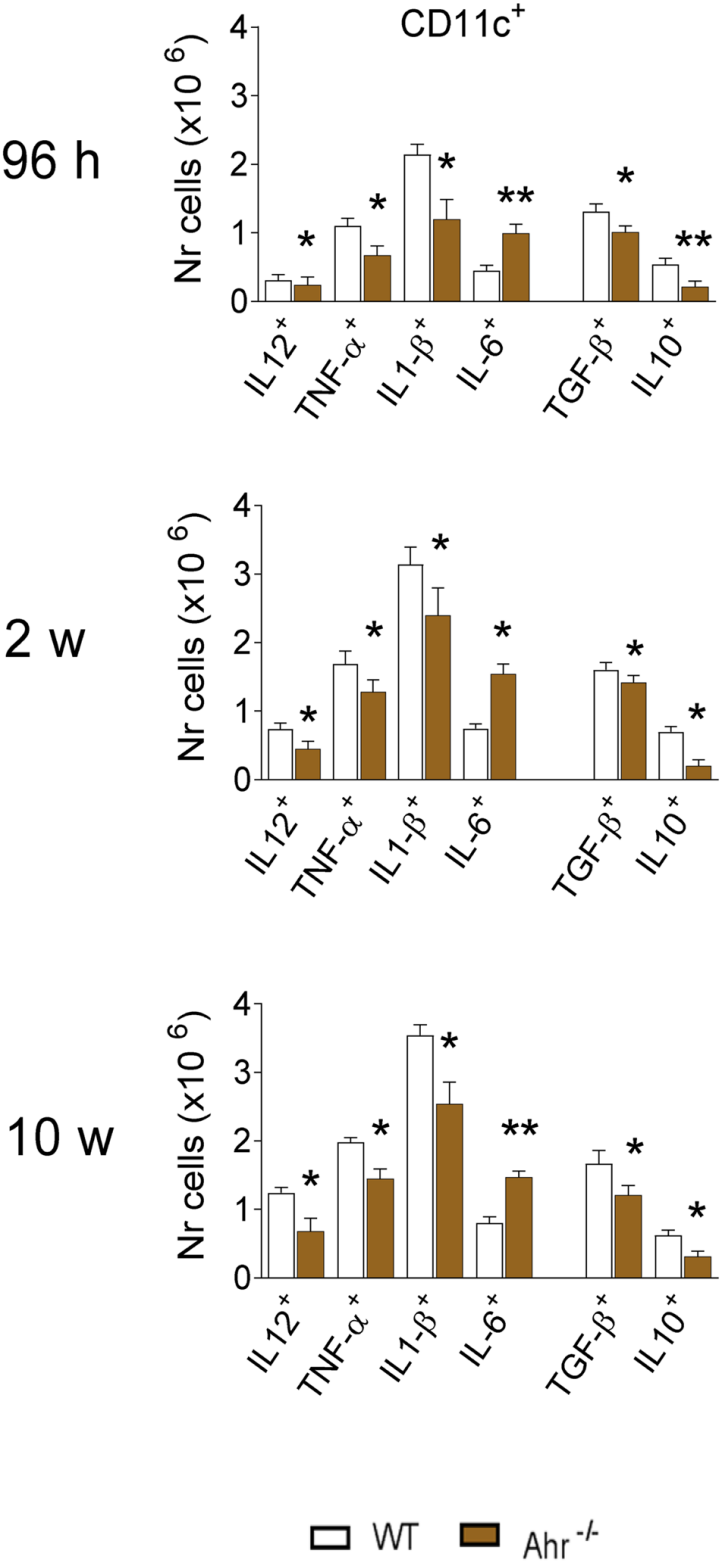
Absence of AhR reduces the number of pulmonary CD11c^+^ cells expressing cytokines, except for IL-6^+^ cells. Expression of cytokines (IL-12, TNF-α, IL-1β, IL-6, TGF-β and IL-10) by CD11c^+^ lung leukocytes of AhR^−/−^ and WT mice infected i.t. with 1 × 10^6^ *P. brasiliensis* yeasts. The presence of cytokines was assessed using fluorochrome labelled specific monoclonal antibodies by flow cytometry at the CD1c^+^ cell gates (see Figure S1). Data represent the mean ± SEM of 3 experiments using 5 animals per group. The asterisk represents a statistically significant difference (*P < 0.05 and **P < 0.01).

After 96 h, 2 and 10 weeks of infection lung leukocytes were obtained from infected AhR^−/−^ and WT mice and analyzed by flow cytometry for the presence of intracellular cytokines (IL-12, TNF-α, IL-1β, IL-6, TGF-β and IL-10) in CD11c^+^ gated cells. A very homogeneous pattern was observed: in AhR^−/−^ mice a reduced number of CD11c^+^ cells expressing pro-inflammatory (IL-12, TNF-a and IL-1b) and anti-inflammatory (TGF-b and IL-10) cytokines was seen at all time points assayed. In contrast, a higher number of CD11c^+^ IL-6^+^cells were found in the lungs of AhR^−/−^ mice that in WT controls (Fig. 3).

#### Absence of AhR decreases the presence of NK, ILC3 and NCR IL-22 innate lymphoid cells (ILCs) in lungs of *P. brasiliensis* infected mice

**Figure 4:**
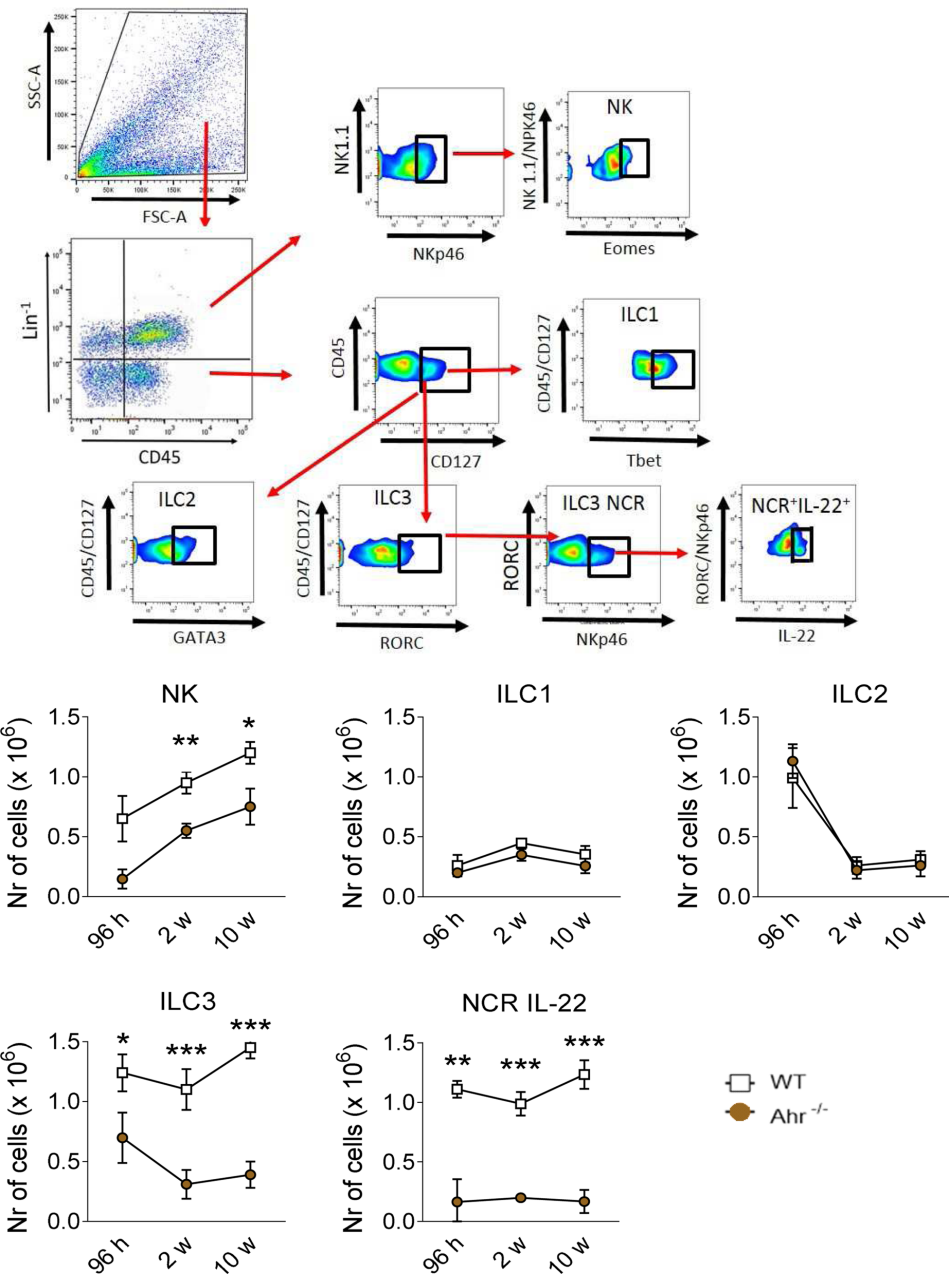
Absence of AhR decreases the presence of NK, ILC3 and NCR IL-22 innate lymphoid cells (ILCs) in the lungs of *P. brasiliensis* infected mice. The phenotypic analysis of ILCs in the lungs of WT and AhR^−/−^ mice was performed after 96 h, 2 and 10 weeks of *P. brasilienis* infection. Lung leukocytes were first treated with anti-mouse lineage cocktail (Biolegend) containing antibodies to CD3, Ly6G/Ly6C, CD11b, CD45R/B220, TER 119/erytroid cells, that react with T cells, B cells, monocytes, macrophages, NK cells and erythrocytes. NK cells were then classified as Lin^+^CD45^+^NK1.1^+^NKp46^+^Eomes^+^, ILC1 as CD45^+^Lin^−^CD127^+^Tbet^+^, ILC2 as CD45^+^Lin^−^CD127^+^Gata3^+^, ILC3 as CD45^+^Lin^-^CD127^+^RORC^+^ and NCR^+^IL-22^+^ as CD45^+^Lin^−^CD127^+^RORC^+^NKp46^+^IL-22^+^. The cell surface and intracellular markers were measured by flow cytometry. 50.000 cells were counted, and the data expressed by number of positive cells. Data are expressed as M ± SEM and are representative of three independent experiments using 5 mice of each mouse strain per group (*P < 0.05; **P < 0.01 and ***P < 0.001).

The expression of AhR is important for the maintenance, survival, and function of ILC3. In addition, AhR cooperates with RORγt to induce IL-22 transcription^25,28,42^, a fact that led us to phenotypically characterize these cells in *P. brasiliensis* infected WT and AhR^−/−^ mice. Therefore, ILC subpopulations were categorized by their expression of specific surface molecules and transcription factors as described in material and methods. NK cells were classified as Lin^+^CD45^+^NK1.1^+^ NKp46^+^Eomes^+^, ILC1 as CD45^+^Lin^-^CD127^+^Tbet^+^, ILC2 as CD45^+^Lin^-^CD127^+^Gata3^+^, ILC3 as CD45^+^Lin^-^CD127^+^RORC^+^ and NCR^+^IL-22^+^ as CD45^+^Lin^-^CD127^+^RORC^+^NKp46^+^IL-22^+^. As can be seen in Fig. 4, in the whole course of the infection a marked decrease in the number of NK, ILC3 and NCR IL-22 lymphocytes was observed in the lungs of AhR^−/−^. No differences in ILC1 and ILC2 were observed between AhR^−/−^ and their WT counterparts (Fig. 4).

### Influence of AhR on gene expression in lungs of *P. brasiliensis* infected mice

**Figure 5:**
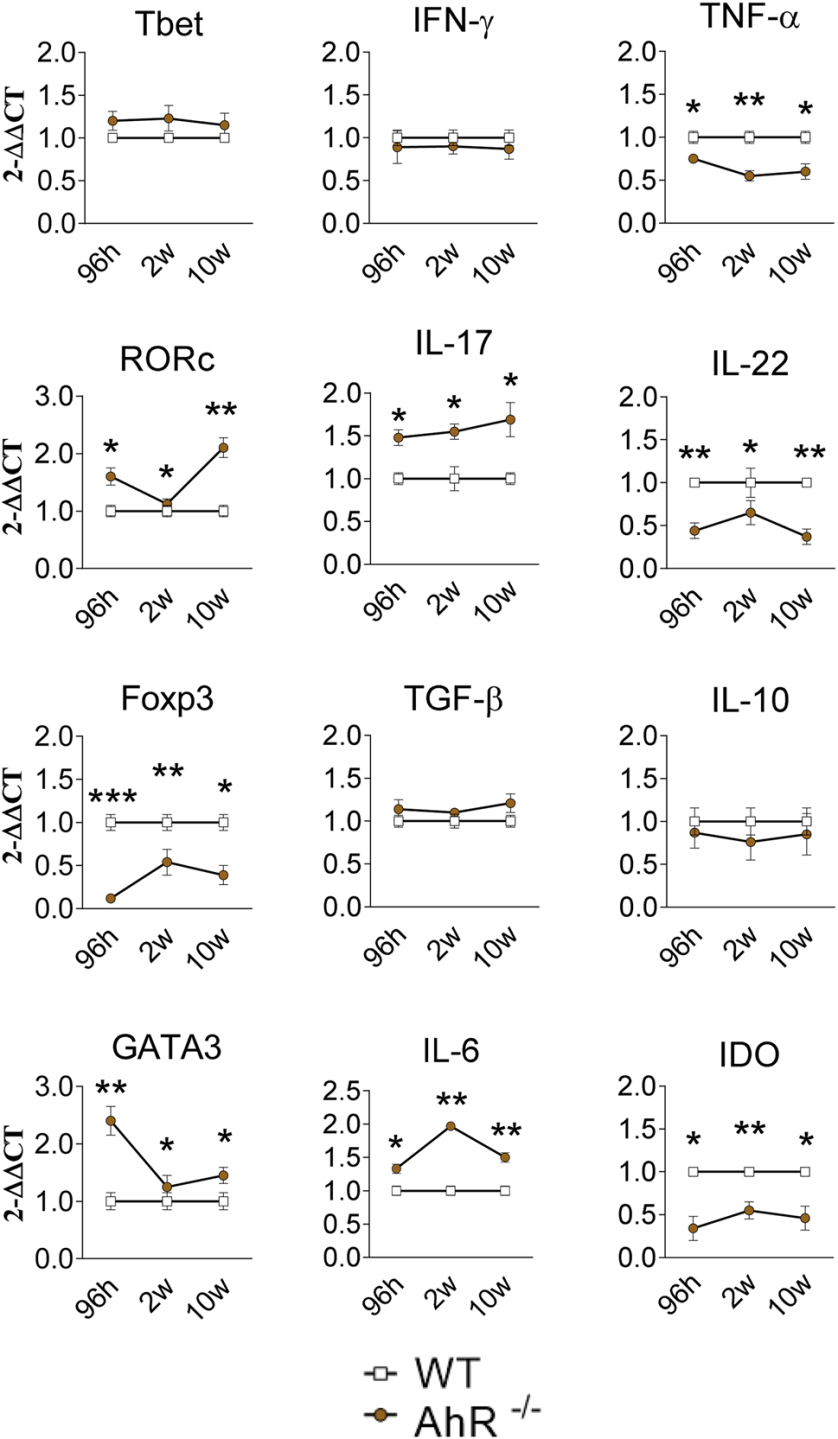
Absence of AhR alters mRNA levels of cytokines, transcription fators and the IDO enzyme. The relative expression of mRNA of pro-and anti-inflammatory cytokines (IFN-γ, TNF-α, IL-6, IL-17, IL-22, TGF-β and IL-10), transcription factors (Tbet, RORc, Foxp3 and GATA-3) as well as the IDO enzyme was determined by Real-Time PCRin total lung cells of AhR^−/−^ and WT mice after 96 h, 2 and 10 weeks of *P. brasiliensis* infection. Data are shown as M ± SEM from three independent experiments using 5 mice per group. The asterisk represents a statistically significant difference (*P < 0.05 and **P < 0.01, ***P < 0.001).

The expression of mRNA for some cytokines (*ifn-γ, tnf-α, il-6, il-17 and il-22*), IDO-1 enzyme (*ido*), and transcription factors (*tbet, gata3, rorc and foxp3*) involved in the immunity and phenotypic differentiation of lymphocytes^43^ was determined in infected AhR^−/−^ and WT mice after 96 h, 2 and 10 weeks of *P. brasiliensis* infection. As shown in Fig. 5, at all-time points assessed no differences in Tbet and IFN-g mRNA expression, markers of Th1 immunity, were seen but TNF-a mRNA was produced in reduced levels by AhR deficient mice. mRNA expression of RORc and IL-17 (Th17 markers) appeared in higher levels in AhR^−/−^ mice; IL-22, however, that is highly dependent on AhR activity was detected in reduced levels in the lungs of AhR deficient mice. Compared with WT controls, AhR^−/−^ mice showed diminished expression of Foxp3 mRNA, but not TGF-b or IL-10, transcription factor and cytokines associated with Treg cells differentiation and activity. GATA3 (a Th2 transcription factor) and IL-6 (a pleiotropic cytokine) mRNAs were produced in higher levels, whereas IDO mRNA expression was lower in AhR^−/−^ mice than in WT controls (Fig. 5).

### Influence of AhR on the production of pulmonary cytokines

**Figure 6:**
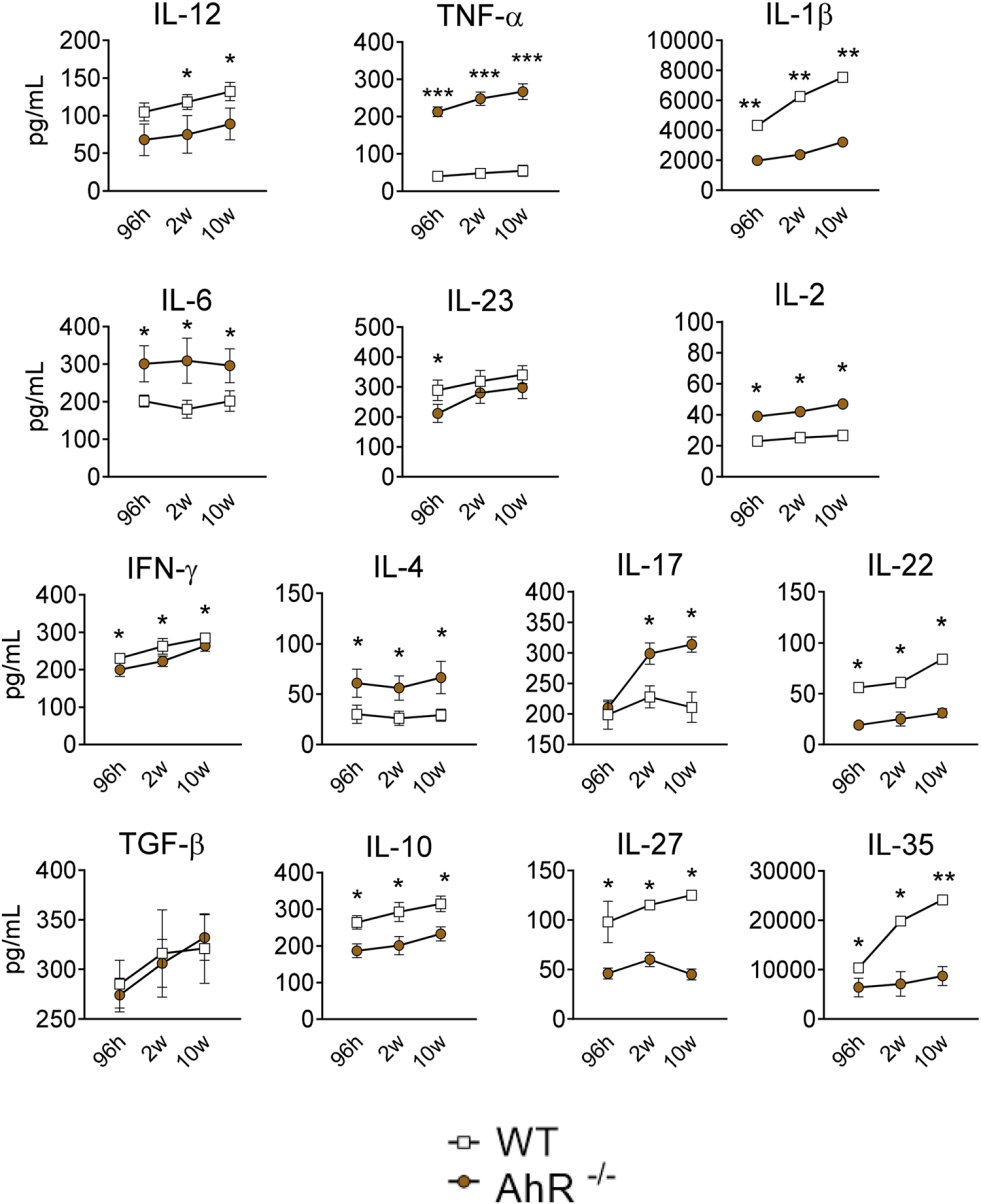
Absence of AhR alters the levels of cytokines in the lung. Pro- and anti-inflammatory cytokines were measured by ELISA in lung homogenates of *P. brasiliensis* infected AhR^−/−^ and WT mice obtained 96 h, 2 and 10 weeks after infection. Data show M ± SDM of one experiment using 5 mice per group. The asterisk represents a statistically significant difference (*P < 0.05 and **P < 0.01, ***P < 0.001).

The lung homogenates of WT and AhR^−/−^ mice were collected after 96 h, 2 and 10 weeks of infection and used to evaluate the presence of cytokines (IL-12, TNF-α, IL-1β, IL-6, IL-23, IL-2, IFN-γ, IL-4, IL-17, IL-22, TGF-β, IL-10, IL-27, and IL-35). As can be seen in Fig. 6, increased levels of TNF-α, IL-6, IL-2, and IL-4 were detected in the lungs of AhR deficient mice at all time points evaluated. IL-17 appeared in higher levels at weeks 2 and 10 following infection. In contrast, IL-1β, IFN-γ, IL-22, IL-10, IL-27 and IL-35 were present in lower levels in the lungs of these mice at all post-infection periods assessed. Interestingly, IL-1β was the pro-inflammatory cytokine most noticeably reduced in the lungs of AhR deficient mice. TGF-β was the only cytokine in the group of suppressive or inhibitory cytokines that was not affected by the absence of AhR expression. All other inhibitory cytokines involved in the development or activity of Tr1 and Treg (IL-27, IL-10 and IL-35) cells were detected in reduced concentrations in the lungs of AhR deficient mice.

### Absence of AhR increases Th17 cells but reduces Th1, Th22 and Treg cells in the lungs of *P. brasiliensis* infected mice

The number and phenotype of T cells that were present the lungs of AhR^−/−^ mice and WT controls were evaluated by flow cytometry at weeks 2 and 10 following infection.

**Figure 7:**
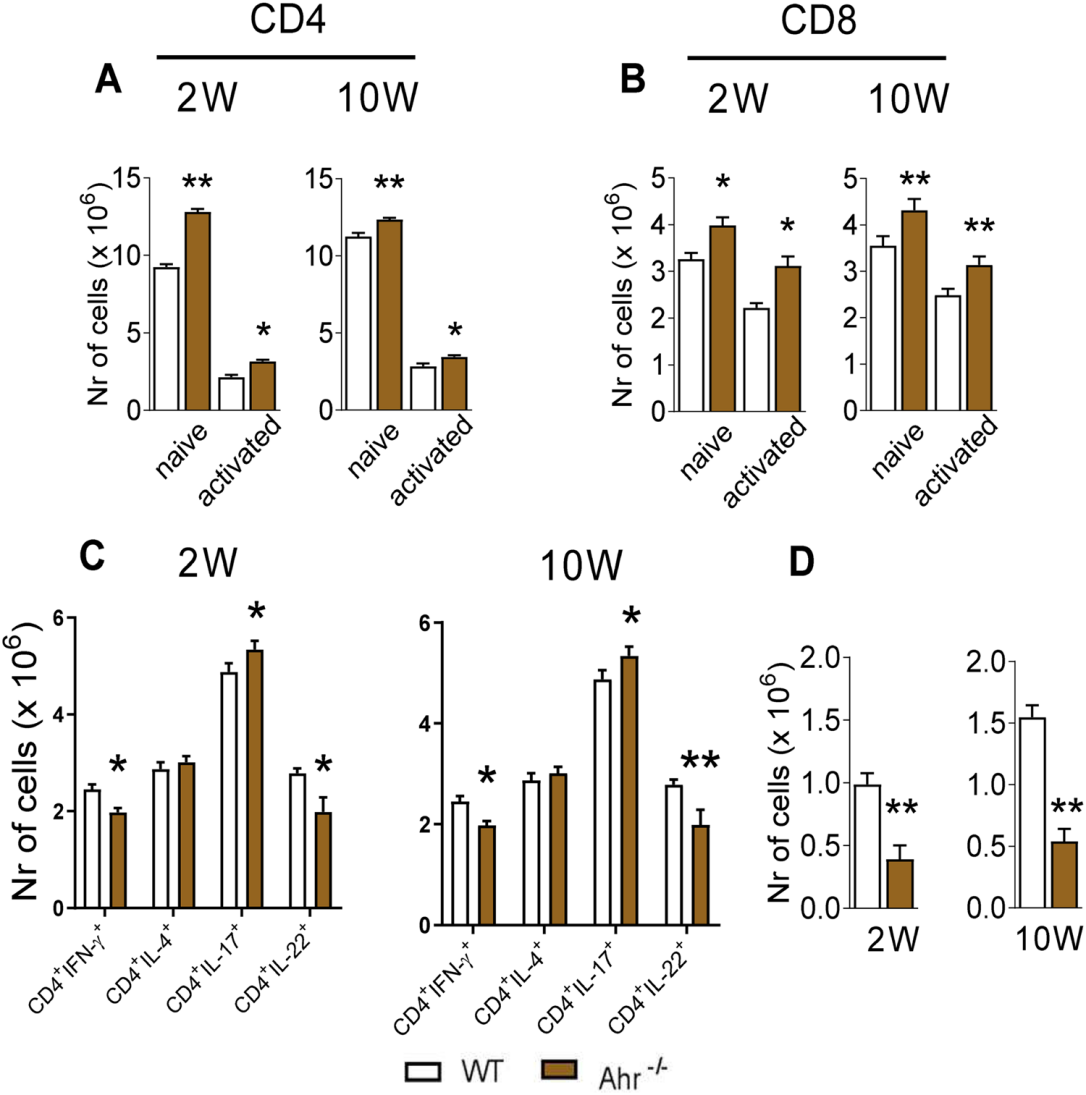
Absence of AhR increases the presence of Th17 cells into the lungs but reduces Th1, Th22 and Treg cells. The lung cells were obtained as described in Material and Methods and labeled with antibodies conjugated to different fluorochromes. For T cells phenotyping the lung infiltrating leukocytes were gated by FSC/SSC analysis and then gated for CD4^+^ or CD8^+^ expression. The expression of CD44 and CD62L was then evaluated in CD4^+^ and CD8^+^ T cells (for gates strategy see figures S2 and S3). (**A**) CD4^+^CD44^low^CD62L^high^ and CD4^+^CD44^high^CD62L^low^ were considered naive and activated CD4 cells, respectively. (**B**) CD8^+^CD44lowCD62L^high^, CD8^+^CD44^high^CD62L^low^ were considered naive and activated CD8 cells, respectively. (**C**) For Th cell subsets, cells were gated for CD4^+^ expression and then for intracellular expression of IFN-γ, IL-4, IL-17 and IL-22 (gate strategy is shown in figure S4 B). (**D**) For Treg cells, CD4 gated cells were then analyzed for CD25 and Foxp3 expression (gate strategy in figure S4 A). 100.000 cells were acquired on FACS CANTO II and subsequently analyzed by FlowJo software. Data are expressed as M ± SEM of three independent experiments using 5 mice per group. The asterisk represents a statistically significant difference (*P < 0.05 and **P < 0.01, ***P < 0.001.

A higher number of naïve and activated CD4^+^ (CD4^+^CD44^low^CD62L^high^ and CD4^+^ CD44^high^CD62L^low^, respectively) T lymphocytes were observed in the lungs of AhR deficient mice at both post-infection periods studied (Fig. 7A). The same was observed for naïve and activated CD8^+^ (CD8^+^CD44^low^CD62L^high^ and CD8^+^CD44^high^CD62L^low^, respectively) T cells (Fig. 7B).

When the Th1, Th2, Th17 and Th22 subpopulations were characterized (Fig. 7C), a reduced number of Th1 cells (CD4^+^IFN-γ^+^) and Th22 (CD4^+^IL-22^+^) cells were found in the lungs of AhR^−/−^ mice in comparison with the WT controls. In contrast, Th17 (CD4^+^IL-17^+^) lymphocytes were observed in increase numbers in AhR deficient mice. When the number of regulatory CD4^+^CD25^+^FoxP3^+^ T lymphocytes (Tregs) was assessed, a significant decrease was detected in the lungs of AhR deficient mice at both time points analyzed (Fig. 7D). No differences were found in Th2 cells (Fig. 7C).

### Treatment of *P. brasiliensis* infected mice with an AhR-specific antagonist (CH223101) reproduces the main findings in AhR^−/−^ mice

**Figure 8:**
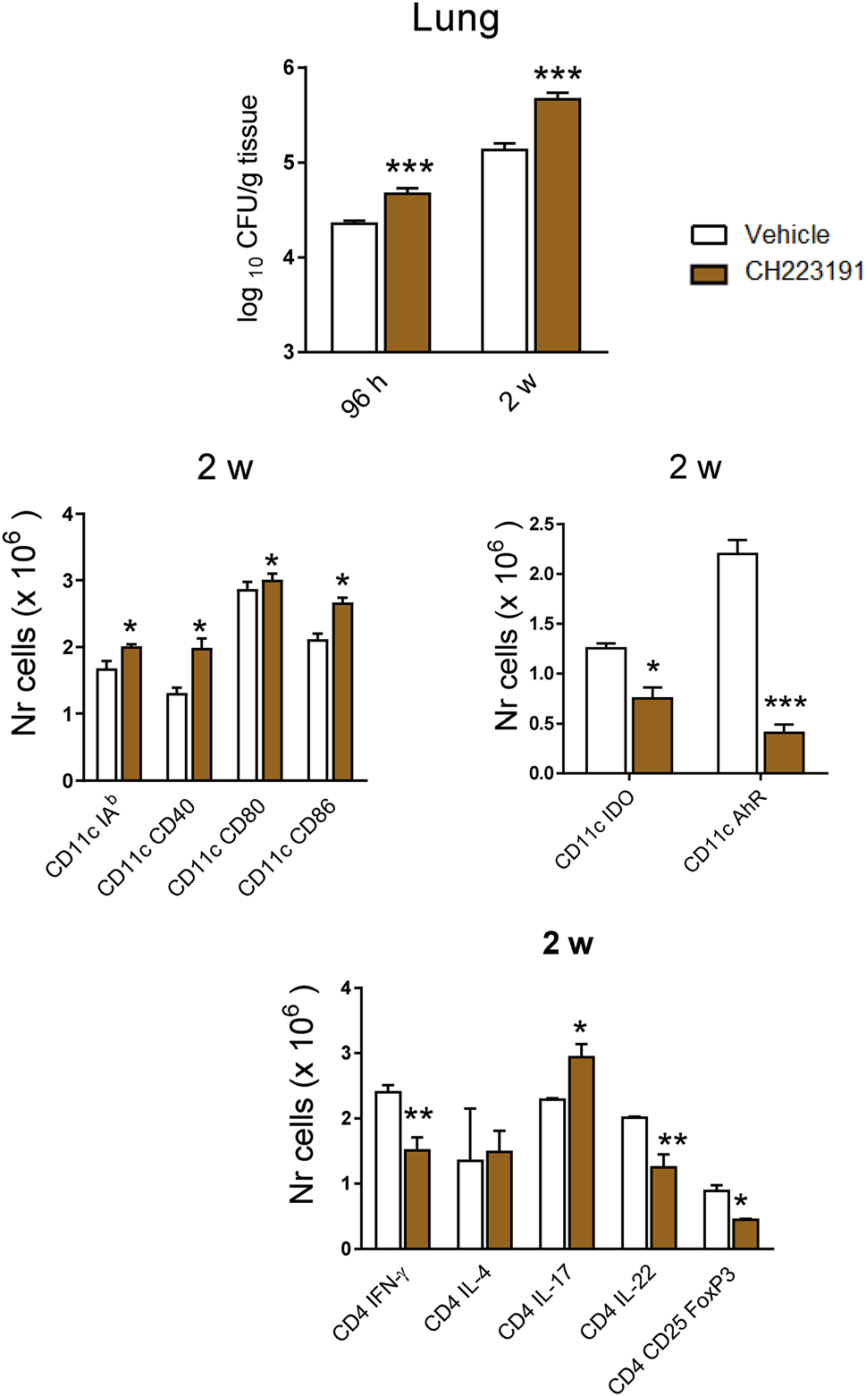
Pulmonary paracoccidioidomycosis can be immunomodulated by an AhR-specific antagonist (CH223191) that reproduces the main findings obtained in AhR-deficient mice. C57BL6/J WT mice were i.p. treated with vehicle (corn oil) or CH223191 at the dose of 20 mg/kg three times a week for 2 weeks, starting at the day of infection with 1 × 10^6^ *P. brasiliensis* yeasts. CFU counts were measured 96 h and 2 weeks after infection. The number of CD11c^+^ cells expressing membrane (IA^b^, CD80, CD86) and intracellular (IDO-1 and AhR) markers as well as the phenotype of CD4^+^ T cell subsets were measured by flow cytometry in lung leukocytes obtained at week 2 of infection. Data are shown as M ± SEM from three independent experiments using 4–5 mice per group. The asterisk represents a statistically significant difference (*P < 0.05 and **P < 0.01, ***P < 0.001).

Finally, we extended the results obtained with AhR^−/−^ mice by assessing the antagonist effects of CH223191 in pulmonary PCM. Mice were treated as described in Material and Methods, infected with 1 × 10^6^ fungal cells and studied at 96 h and 2 weeks after infection. Treatment with CH223191 increased the fungal load at both post-infection periods studied (Fig. 8). At week 2, CD11c^+^ leukocytes showed increased numbers of MHC class II molecules (IA^b^), CD40, CD80 and CD86. However, the number of CD11c^+^ cells expressing IDO-1 or AhR was significantly reduced. Characterizing the main T cell phenotypes present in the lungs of CH223191-treated mice, an augmented number of Th17 cells was concomitant with a reduction in Th1, Th22 and Foxp3^+^ Treg cells (Fig. 8). These findings reproduce the main phenotypes observed in AhR^−/−^ mice, further evidencing the immunomodulatory activity of AhR in pulmonary PCM.

## Discussion

**Figure 9:**
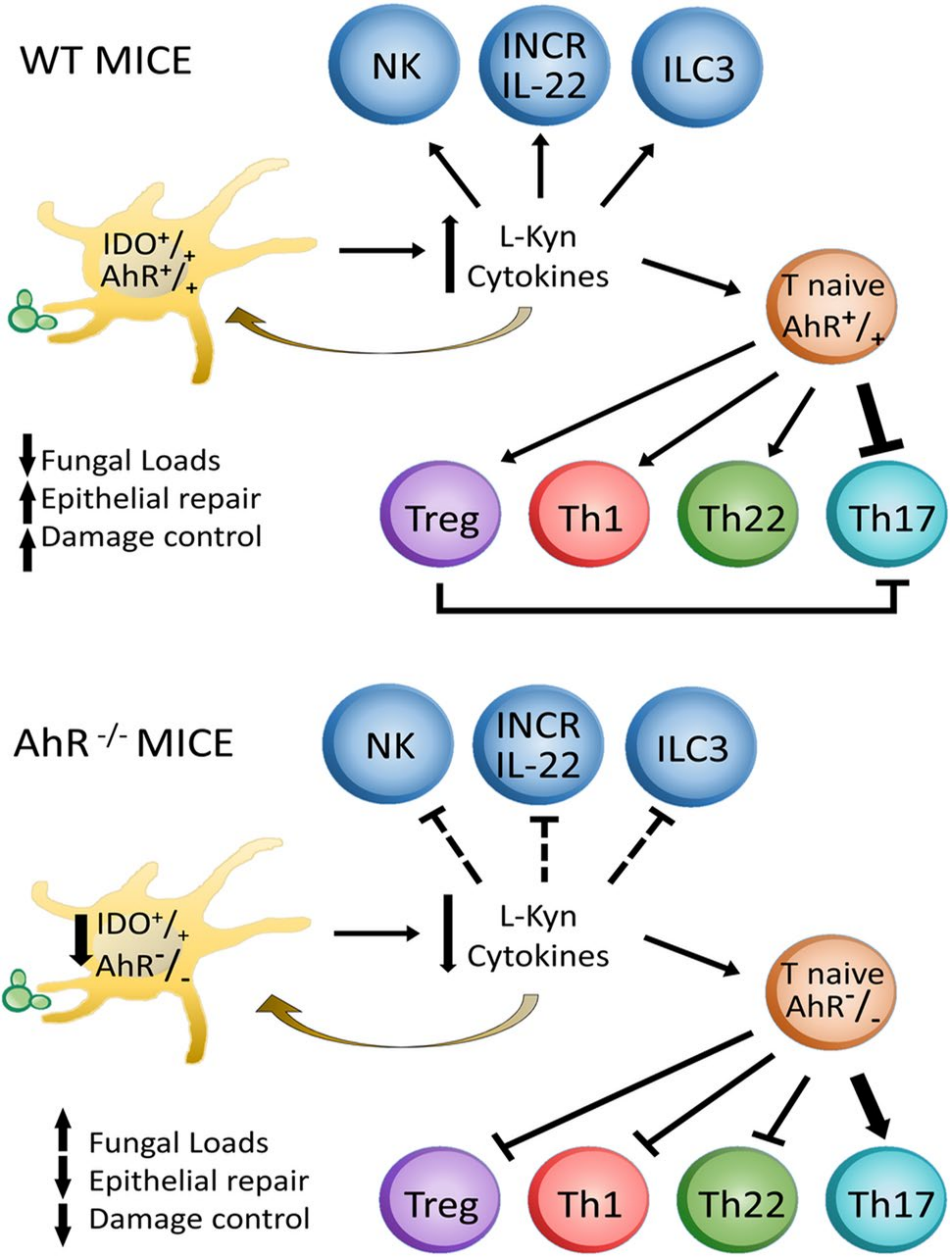
Schematic representation of AhR deficiency in the innate and adaptive phases of immunity against *P. brasiliensis* infection. Compared with control WT mice (**A**), AhR^−/−^ mice develop a defective innate and adaptive immunity (**B**). Absence of AhR expression alters the behavior of DCs that reduce IDO-1 expression and produce low levels of Kynurenines (Kyn). AhR deficiency also impairs the development of NK, ILC3 and NCR-IL-22 innate lymphoid cells involved in the synthesis of IFN-γ, IL-17 and IL-22 at the innate phase of immunity. At the adaptive phase of immunity, the reduced AhR activity results in decreased differentiation and expansion of Treg/Th1/Th22 cells but an increased proliferation of Th17 cells. In AhR^−/−^ mice, the reduced IDO-1 activity allows high tryptophan availability that enhances fungal growth not adequately controlled by pro-inflammatory Th17 cells that possibly contribute with tissue pathology and increased mortality observed in deficient mice.

Once considered a mediator in the toxic response to dioxin, the AhR was subsequently described as an important regulator of the immune response, including the immunity against infectious agents^12^. Besides the induction of detoxifying enzymes, AhR modulates the differentiation and activity of innate and adaptive immune cells^9,10^, markedly influencing the outcome of infectious processes. Indeed, here we could demonstrate that AhR has an important regulatory role in pulmonary PCM. The Fig. 9 summarizes the main findings of AhR deficiency on the innate and adaptive phases of immunity in *P. brasiliensis* infected mice. The lack of AhR expression reduces cytokines, IDO-1 expression and Kyn synthesis by pulmonary DCs. This altered pulmonary microenvironment leads to defective innate immunity mechanisms with reduced participation of NK, ILC-3 and NCR-IL-22 cells. At the adaptive phase of immunity that subsequently develops, the lack of AhR leads to reduced expansion of the Th1, Th22 and Treg cells associated with increased Th17 development, whose pro-inflammatory activity is only partially controlled by the reduced number of Treg cells. The diminished IDO activity also promotes increased Trp availability that facilitates fungal growth. Altogether, the increased fungal loads and the inefficient effector mechanisms of immunity possibly contribute to the enhanced pulmonary pathology and elevated mortality rates developed by AhR^−/−^ mice. It is important to comment that the inclusion of additional controls showing base lines differences between WT and AhR deficient mice would improve data analysis. However, the limitations imposed by the difficulty of obtaining a sufficient number of AhR^−/−^ mice, as most fetuses do not develop due to the serious genetic errors associated with this deficiency, and the restriction to the use of large number of animals imposed by the ethics committee for animal use justify the experimental design here employed. Nevertheless, the differences here observed were probably due to the genetic differences associated with AhR deficiency, since both AhR^−/−^ and WT mice belong to the same C57BL/6 strain. Furthermore, experiments with the AhR antagonist reproduced the main findings observed in AhR deficient mice, indicating that the different responses observed were linked to AhR expression.

An important finding in AhR biology was the mutual control exerted by AhR and IDO-1 expression^44–46^, a finding here confirmed in pulmonary PCM. This enzyme has been studied for its antimicrobial ability mainly mediated by Trp starvation^47^ and also by the immunoregulatory activity of kynurenines^48–50^. In fungal infections, the importance of the IDO-1/Kyn/AhR axis in the generation of Treg cells and tolerogenic activities of DCs was demonstrated^37,51,52^. In pulmonary PCM, our group has shown a profound interconnection between IDO-1 and AhR expression where a balanced activation of these mediators was connected with the control of fungal immunity and disease tolerance^36–39^.

Fundamental studies have revealed that AhR controls several mechanisms of immunity, due to its influence in innate immunity cells (ILC3, LTi, NCR IL-22, macrophages, DCs) and cells of adaptive immunity (Treg, Th17, Th22)^8–10^. Here we could demonstrated that in pulmonary PCM the lack of AhR expression modified the innate and adaptive phases of immunity resulting in increased fungal burden, increased tissue pathology and mortality rates of *P. brasiliensis* infected mice. Analogous results were observed in *Listeria monocytogenes* but not in *Trypanosoma cruzi* infected AhR^−/−^ mice. Interesting, in *T. cruzi* infection, an increased NO production by AhR-deficient mice was concomitant with reduced pathogen loads^53^, an observation not reproduced in our study. Despite the increased NO levels detected in AhR^−/−^ mice, an increased fungal burden was observed, suggesting that in pulmonary PCM the Trp depletion mediated by the enzymatic activity of IDO-1 exerts a more efficient fungicidal mechanism than that mediated by NO.

The lack of AhR resulted in decreased numbers of pulmonary CD11c^+^ cells expressing pro-inflammatory (IL-12, TNF-α and IL-1β) and anti-inflammatory (TGB-b and IL-10) cytokines, except for IL-6 producing cells for which were found in increased numbers. This reduction in cytokines production occurs despite the increased numbers of CD11c^+^ cells expressing activation markers (IA^b^, CD80, CD86). However, this diverse activation profile was possibly involved in the reduced expansion of some T cell subsets (Th1, Th22, Treg) and the increased proliferation of others (Th17). In agreement, IL-6 that is a positive regulator of Th17 cells via STAT3 activation^8–10^, may have contributed to the increased expansion of Th17 cells detected in AhR^−/−^ mice. In addition, AhR-deficient mice have also a significant reduction in the number of CD11c^+^IL-1β^+^ cells as well as in the levels of IL-1β present in lung homogenates. In pulmonary PCM the activation of NLRP3 inflammasome and IL-1β synthesis play an important role in the innate and adaptive phases of immunity^54–57^, therefore the reduction in IL-1β could have contributed to the reduced fungicidal ability of macrophages and DCs but not to the enhanced Th17 activation here described. Hughes et al.^58^ have elegantly demonstrated that IL-1b deficiency is associated with reduced expansion of IL-22^+^NKp46^+^ (NCR IL-22) cells. They showed that conventional DC-derived IL-1β preserves and expands IL-1R1^hi^ IL-22^+^AhR^+^ immature NK cells, potentially influencing mucosal innate immunity during infection. The reduced presence of NCR IL-22^+^ cells here detected may be associated with the deficient IL-1β production but the absence of AhR, that is fundamental to the expansion of IL-22 producing cells^59,60^, was possibly the main mechanism responsible for the reduced numbers of NCR IL-22^+^ cells observed. The lungs of AhR^−/−^ mice presented lower number of CD11c^+^TGF-β^+^ cells than did WT mice. TGF-β plays an important role in the differentiation of Th17 and Treg cells^61^ and the reduced synthesis of TGF-β and IL-1β by AhR^−/−^ mice was unexpectedly accompanied by the enhanced secretion of IL-17, elevated number of Th17 cells and increased RORγt mRNA expression. These findings indicate that increased Th17 associated markers could have been positively modulated by the increased IL-6 production and STAT3 signaling as previously demonstrated^8–10^. In addition, the increased Th17 expansion could be attributed to the increased fungal loads detected in AhR^−/−^ mice.

The reduced presence of myeloid cells expressing intracellular IL-10 and TGF-β and the diminished levels of IL-10 and IL-35 detected in lung supernatants of AhR^−/−^ mice were concomitant with a potent reduction in the number of CD4^+^CD25^+^Foxp3^+^ Treg cells, possibly explaining the increased lung inflammation mediated by the elevated presence of Th17 cells. In addition, the low levels of IL-10 and IL-27 could also be linked to a diminished number of IL-10-producing Tr1 cells (not here characterized) that may influence the Treg and Th17 cells^62–64^.

At the 2nd and 10th weeks of *P. brasiliensis* infection a reduced number of conventional NK, ILC3 and NCR IL-22 ILCs were found in the lungs of infected AhR. These findings are in partial agreement with the CD4^+^ T cell subsets and mRNA for cytokines and transcription factors observed in the AhR-deficient animals. Indeed, the reduced NCR IL-22, Th22 and *IL-22* mRNA detected in AhR^−/−^ mice clearly demonstrated that in pulmonary PCM IL-22 production is dependent on AhR expression. On the other hand, the increased Th17 and *RORC* mRNA here characterized suggest that the Th17 phenotype is highly dependent on the *RORC* transcription factor^9^. In contrast, the reduced numbers of ILC3 at the site of infection of AhR^−/−^, showed that these cells are much more dependent on AhR activity than Th17 cells^25,26^.

Regarding adaptive immunity, AhR^−/−^ mice showed an increased differentiation of Th17 cells concomitant with reduced expansion of Foxp3^+^ Treg cells. This finding could be correlated with the reduced IDO-1 expression that in the pulmonary model of PCM was seen to control tolerogenic DCs and Treg cells proliferation^36–39^. On the other hand, contrasting the data with IDO-1^−/−^ mice, a reduced number of CD4^+^IL-22^+^ cells were detected in AhR^−/−^ mice. This result also agrees with the lower presence of IL-22 in lung homogenates of the AhR^−/−^ mice, the reduced *IL-22* mRNA expression and the significant reduction of IL-22-secreting ILC3 cells (NCR IL22^+^ cells). Altogether, these data demonstrate that in pulmonary PCM IL-22 synthesis is highly dependent on the AhR transcription factor and, similarly with other fungal infections^65,66^, IL-22 possibly exerts a protective role in pulmonary PCM.

Our previous studies have shown that Treg cells can be protective or deleterious to *P. brasiliensis* infected mice^34,35,67,68^. At all post-infection times assayed, a reduced presence of Treg cells was here observed in line with reduced *Foxp3* mRNA detected and the low levels of IL-10, TGF-β and IL-35, cytokines involved in the suppressive activity of this T cell subpopulation. Interestingly, the reduced TGF-β expression by myeloid cells and not the levels TGF-β mRNA of total lung cells were associated with the decreased Treg cells expansion that was insufficient to control lung inflammation.

Altogether, our data led us to suppose that the decreased presence of NK, ILC3 and NRC IL-22 ILCs, concomitant with the reduced proliferation of Th1 and Th22 cells have possibly affected the IFN-γ mediated fungicidal mechanisms of phagocytes and the IL-22-mediated production of anti-microbial peptides by epithelial cells that play an important role in host defenses in fungal infections^21,22,39,65,66^. Furthermore, these inefficient effector mechanisms appeared to have been potentiated by the decreased presence of Treg cells and their inhibitory effect on TH17-mediated tissue pathology.

Our data using a specific AhR antagonist (CH223191) confirmed the important immunoregulatory role of AhR signaling in the control of pulmonary PCM. Similar to AhR-deficient mice, CH22319-treated WT mice presented increased fungal loads, increased numbers of myeloid cells expressing costimulatory molecules but reduced numbers containing IDO-1 or AhR proteins. In addition, an increased number of Th17 cells was concomitant with reduced presence of Th1, Th22 and Foxp3^+^ Treg cells in the lungs of treated mice. These data suggest that pulmonary PCM can be modulated by AhR ligands and are in agreement with those obtained in experimental vulvovaginal candidiasis where anti-fungal immunity and disease severity can be modulated by an AhR agonist via the IL-22-IL-18 crosstalk^65^.

In conclusion, the data here reported and those previously published by our group^36–39,68^ allowed us to identify the importance of the IDO1/AhR axis in the control Treg/Th17/Th22 development and severity of pulmonary PCM. The absence of AhR expression unbalanced the innate and adaptive phases of immune responses resulting in a deleterious effect to the hosts where an unrestrained fungal growth is associated with uncontrolled inflammatory processes that result in tissue pathology. However, the data presented here open interesting immunotherapeutic perspectives, since the potent immune regulatory activity of AhR may be tentatively used alone or as adjunctive therapy to antifungals to modulate the immune response against *P. brasiliensis* infection and disease severity. We believe that immunomodulatory procedures are of actual importance, since treatment of PCM with antifungals is long lasting and has important side effects that lead to the non-adherence of patients and disease recurrence.

## Methods

### Ethics statement

The experiments were performed in strict accordance with the Brazilian Federal Law 11,794 establishing procedures for the scientific use of animals, and the State Law establishing the Animal Protection Code of the State of São Paulo. All efforts were made to minimize animal suffering. The procedures were approved by the Ethics Committee on Animal Experiments of the Institute of Biomedical Sciences of University of São Paulo (Proc.180/11/CEEA).

### Mice

Eight- to 12-week-old male C57BL6/J WT and B6.129-*Ahr*^*tm1Bra*^/J (AhR^−/−^ mice)^69^ from Jackson Laboratories, originally provided by Dr. Marc Veldhoen and bred as specific pathogen free mice at the Isogenic Breeding Unit of the Department of Immunology, Institute of Biomedical Sciences, University of São Paulo, were used throughout this study.

### Fungus and infection

Yeast cells from the virulent *P. brasiliensis *18 isolate (Pb18) was maintained by weekly cultivation in Fava Netto culture medium at 36 °C and used on days 6–8 of culture. The viability of fungal cells, determined by Janus Green B vital dye (Merck), was always higher than 95%. Mice were anesthetized and submitted to intra-tracheal (i.t.) infection as previously described^70^. Briefly, after intraperitoneal (i.p.) injection of ketamine and xylazine, animals were infected with 1 × 10^6^ yeast cells, contained in 50 µL of PBS, by surgical i.t. inoculation, which allowed dispensing of the fungal cells directly into the lungs.

### CFU assays, mortality rates, and histological analysis

The number of viable yeasts in lung and liver was determined by counting the number of colony-forming units (CFU) as previously described^71^. Mortality studies were done with groups of 10–12 mice. Deaths were registered daily. For histological examinations, five-micrometer tissue sections were stained by hematoxylin–eosin for characterization of lesions and were silver stained (Grocott stain) for fungal evaluation. Morphometrical analysis was performed using a Nikon DXM 1200c camera and Nikon NIS AR 2.30 software. The areas of lesions were measured (in square micrometers) in 10 microscopic fields per slide in 5 mice per group as previously described^72^. Results are expressed as the mean ± SEM of total area of lesions for each mouse.

### Assessment of leukocyte subpopulations and intracellular cytokines by flow cytometric analysis

The lungs from *P. brasilienis* infected WT and AhR^−/−^ mice were collected after 96 h, 2 and 10 weeks of infection, digested enzymatically and lung leukocytes prepared as previously described^39^. For cell-surface staining, lung cells were suspended at 1 × 10^6^ cells/mL in staining buffer. Fc receptors were blocked with unlabeled anti-CD16/32 (eBioscience) and then stained for 30 min on ice with fluorophore-conjugated antibodies. For myeloid cells the following antibodies were used: anti-CD11c, CD40, CD80, CD86 and MHC-II (IAb^+^). For lymphocytes: anti-CD4, CD25, CD8, CD44, and CD62L. For ILCs characterization, lung leukocytes were first treated with anti-mouse lineage cocktail (Biolegend) containing antibodies to CD3, Ly6G/Ly6C, CD11b, CD45R/B220, TER 119/erytroid cells, that react with T cells, B cells, monocytes, macrophages, NK cells and erythrocytes. Intracellular staining was conducted using the eBioscience Transcription Factor staining kit and specific antibodies for IL-17, IL-4, IFN-γ, IL, 22, IL-1β, IL-12, TNF-α, IL-6, TGF-β, IL-10, FoxP3, IDO-1 and AhR. Cells were run on FACSCantoII (BD Biosciences) and a minimum of 50,000 events was acquired using a FACSDiva software (BD Biosciences). Cells were analyzed using FlowJo software (Tree Star).

### RNA isolation, cDNA synthesis and Real-Time quantitative polymerase chain reaction (RT–PCR)

RNA isolation from lungs of AhR^−/−^ and control mice was done as previously described^39^. RNA purity and concentration were assessed on a NanoDrop ND-1000 spectrophotometer. An amount of 1 μg total RNA was reverse transcribed in a 20 μL reaction mixture using the High Capacity RNA-to-cDNA kit (Applied Biosystems) following the manufacturer’s directions. The cDNA was amplified using TaqMan Universal PCR Master Mix (Applied Biosystems) and pre-developed TaqMan assay primers and probes (*Ifng*, Mm001168134_m1, *Tnf,* Mm99999068_m1, *Il6,* Mm00446190_m1, *Il10,* Mm00439614_m1, *Tgfb1,* Mm00117882_m1, *Il17,* Mm00439618_m1, *Il22,* Mm01226722_m1, *Tbet*, Mm00450960_m1; *Gata3*, Mm00484683_m1; *Rorc*, Mm01261022_m1; *Foxp3*, Mm00475162_m1; *Gapdh*, Mm99999915_g1a, ll from Applied Biosystems). PCR assays were performed on an MxP3000P QPCR System and data were developed using the MxPro qPCR software (Stratagene). The average threshold cycle (CT) values of samples were normalized to CT value of *Gapdh* gene. The relative expression was determined by the 2^-ΔΔ^CT method.

### Cytokines and NO detection

After 96 h, 2 and 10 weeks of infection, the lungs from *P. brasilienis*-infected WT and AhR^−/−^ mice were aseptically collected, disrupted, and the obtained supernatants were stored at − 80 °C. Cytokines and NO levels were measured as previously described^39^. A capture ELISA (enzyme-linked immunosorbent assay) was used to measure the levels of IL-12, TNF-α, IL-1β, IL-6, IL-23, IL-27, IL-2, IFN-γ, IL-4, IL-17, IL-22, TGF-β, IL-10 and IL-35 with antibody pairs from eBioscience or PBL. Nitric oxide production was quantified by a standard Griess reaction using a spectrophotometric plate reader (VersaMax, Molecular Devices). All determinations were performed in duplicate, and results were expressed as micro molar concentration of NO.

### Treatment of mice with CH223291, an AhR-specific antagonist

Mice were treated with the AhR antagonist CH223191 (InvivoGen) as described by Kim et al.^73^ with minor modifications. In brief, male C57BL6/J WT mice were given vehicle (corn oil) or CH223191 (InvivoGen, 2-methyl-4-o-tolylazo-phenyl-amide) at the dose of 20 mg/kg in corn oil three times a week for 2 weeks, starting at the day of infection. Mice were analyzed after 96 h and 2 weeks of infection.

### Statistical analysis

Data were analyzed as previously described^68^ and expressed as the M ± SEM. Differences between groups were analyzed by non-paired Student's *t* test or analysis of variance (ANOVA) followed by the Tukey test. Survival times differences were characterized using a LogRank test. Data were analyzed using GraphPad Prism 7.03 software (GraphPad Prism Software, Inc.). *p* values ≤ 0.05 were considered significant.

## Supplementary information

Supplementary file1 (PDF 340 kb)

## Data Availability

The datasets generated during and/or analyzed during the current study are available from the corresponding author on reasonable request.
