# Supplementary material for: Pulmonary paracoccidioidomycosis in AhR deficient hosts is severe and associated with defective Treg and Th22 responses
Source: Sci Rep. 2020 Jul 9;10:11312. doi: 10.1038/s41598-020-68322-6 (PMC7347857; doi:10.1038/s41598-020-68322-6)
Supplement: Supplementary file 1 — Supplementary file1 (PDF 340 kb) [file 41598_2020_68322_MOESM1_ESM.pdf]

SUPPLEMENTARY INFORMATION

Pulmonary Paracoccidioidomycosis in AhR deficient hosts is severe and associated with defective Treg and Th22 responses

Eliseu Frank de Araújo[1], Nycolas Willian Preite[1], Marc Veldhoen[2], Flávio Vieira Loures[1, 3] and Vera Lúcia Garcia Calich[1*]

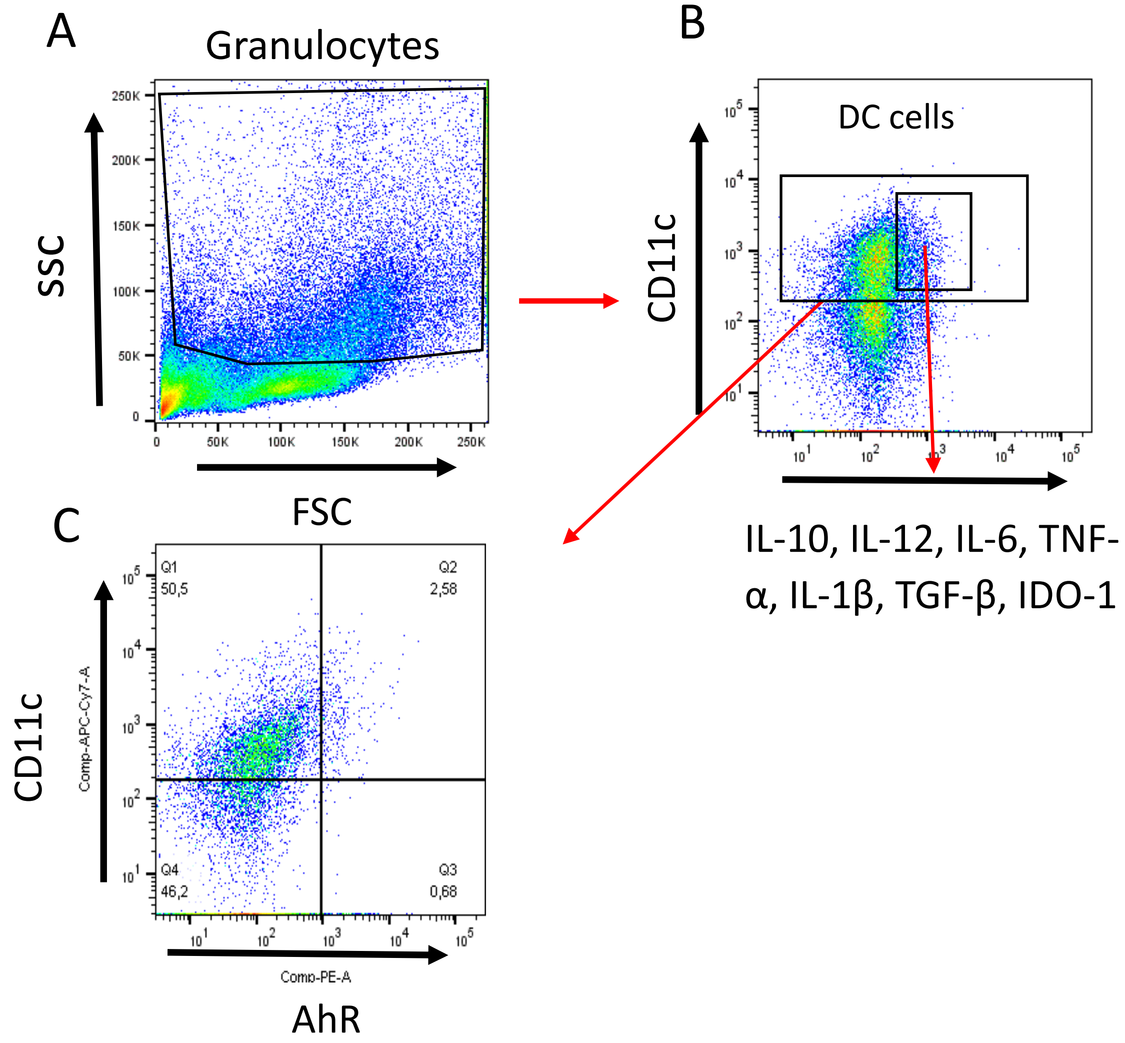

S1 Fig Gate strategy used to characterize CD11c+ granulocytes expressing intracellular cytokines, IDO-1 or AhR. **A-** Lung infiltrating leukocytes were obtained at several post-infection periods, granulocytes gated by SSC and FSC scatters. **B-** Cells were then gated for the expression of membrane CD11c and then for the presence of intracellular cytokines (IL-10, IL-12, IL-6, TNF-a, IL-1b or TGF-b) or the enzyme IDO-1. **C-** Gate strategy used to characterize $CD11c^{+}/AhR^{+}$ cells using APC/Cy7 anti-mouse CD11c (BioLegend, Cat.#117323) and PE anti-AhR (eBioscience, Cat,# 125925-82.

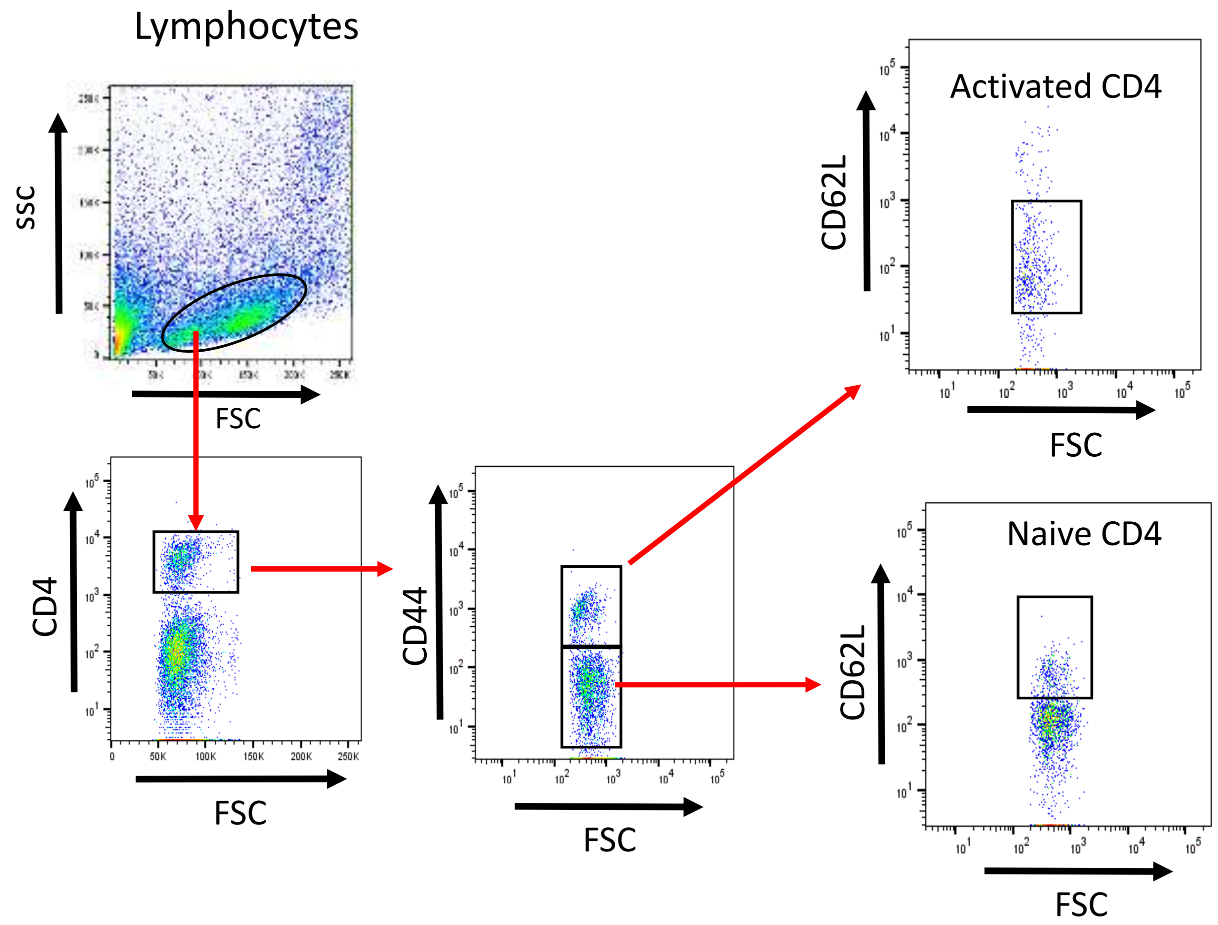

S2 Fig Gate strategy used to characterize naïve and activated CD4^{+} T cells. At several post-infection periods, lung cell suspensions were obtained and stained as described in Materials and Methods. The acquisition and analysis gates were restricted to lymphocytes. The total numbers of effector (CD4^{+}CD44^{high}CD62L^{low}) and naïve (CD4^{+}CD44^{low} CD62L^{high}) CD4^{+} T cells were then determined.

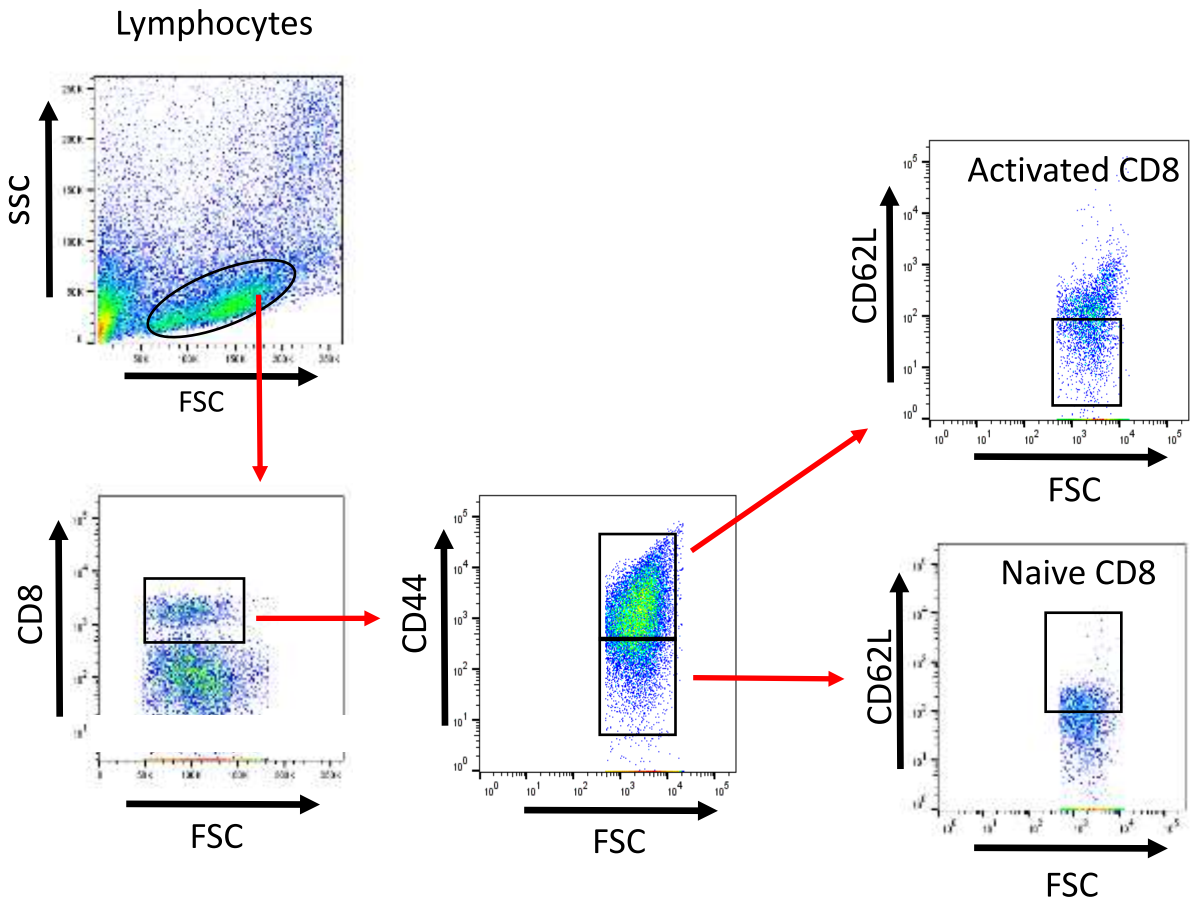

S3 Fig Gate strategy used to characterize naïve and activated CD8$^+$ T cells. At several post-infection periods, lung cell suspensions were obtained and stained as described in Materials and Methods. The acquisition and analysis gates were restricted to lymphocytes. The total numbers of effector (CD8$^+$CD44^{high}CD62L^{low}) and naïve (CD8$^+$CD44^{low} CD62L^{high}) CD8$^+$ T cells were then determined.

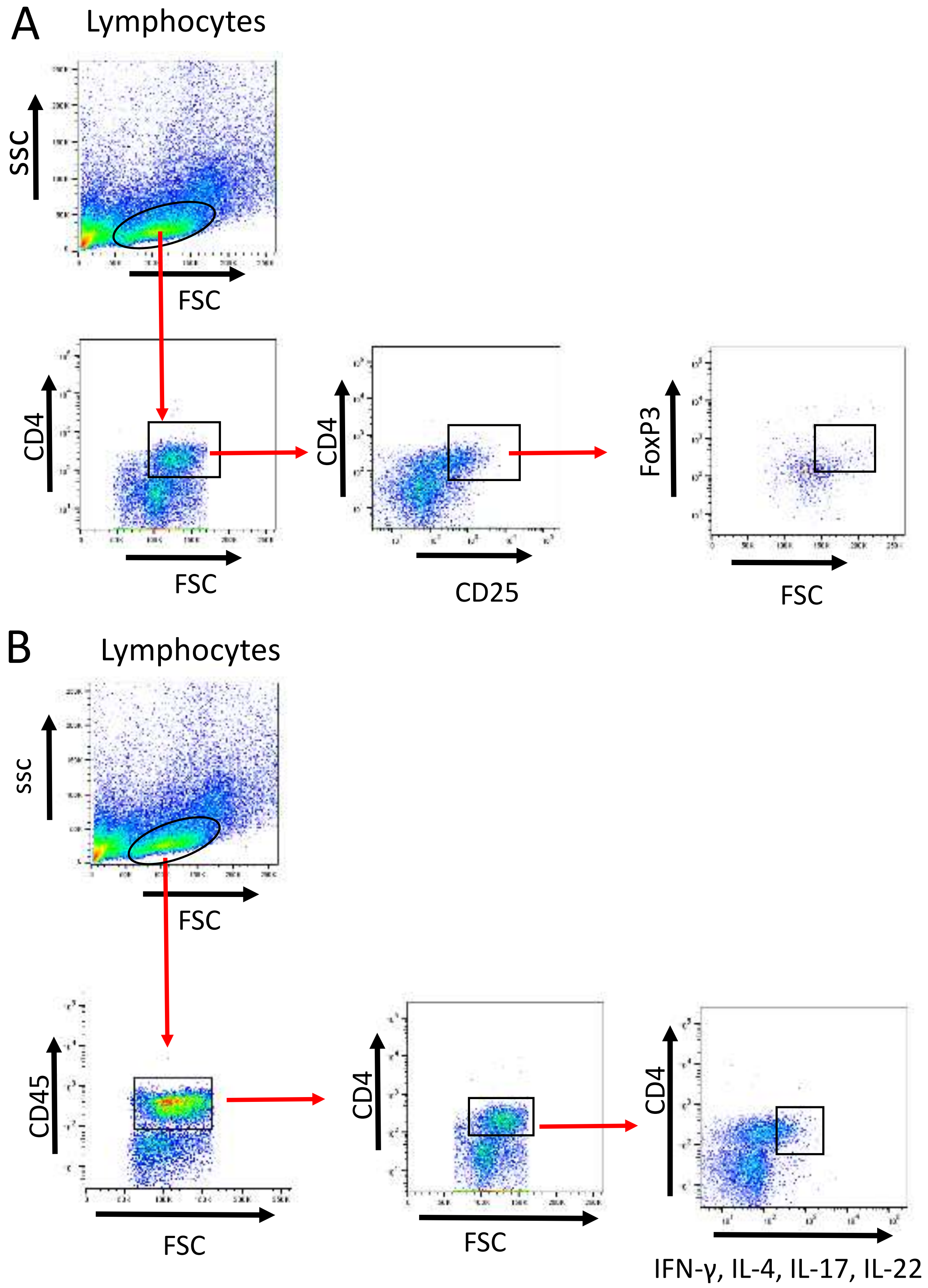

S4 Fig Gate strategy used to characterize Treg cells and intracellular cytokines (IFN-γ, IL-4, IL-17, IL-22) expressed by Th1, Th2, Th17 and Th22 $CD4^+$ T cells. **A-** To characterize the number of Treg cells in lung infiltrating lymphocytes at several periods after

infection, lymphocyte gated cells where then selected for the expression of surface CD4 and CD25 and then for the presence of intracellular Foxp3. **B-** The lymphocyte gated cells were selected for surface CD4 expression and then gated for the presence of intracellular cytokines (IFN-γ, IL-4, IL-17 or IL-22).

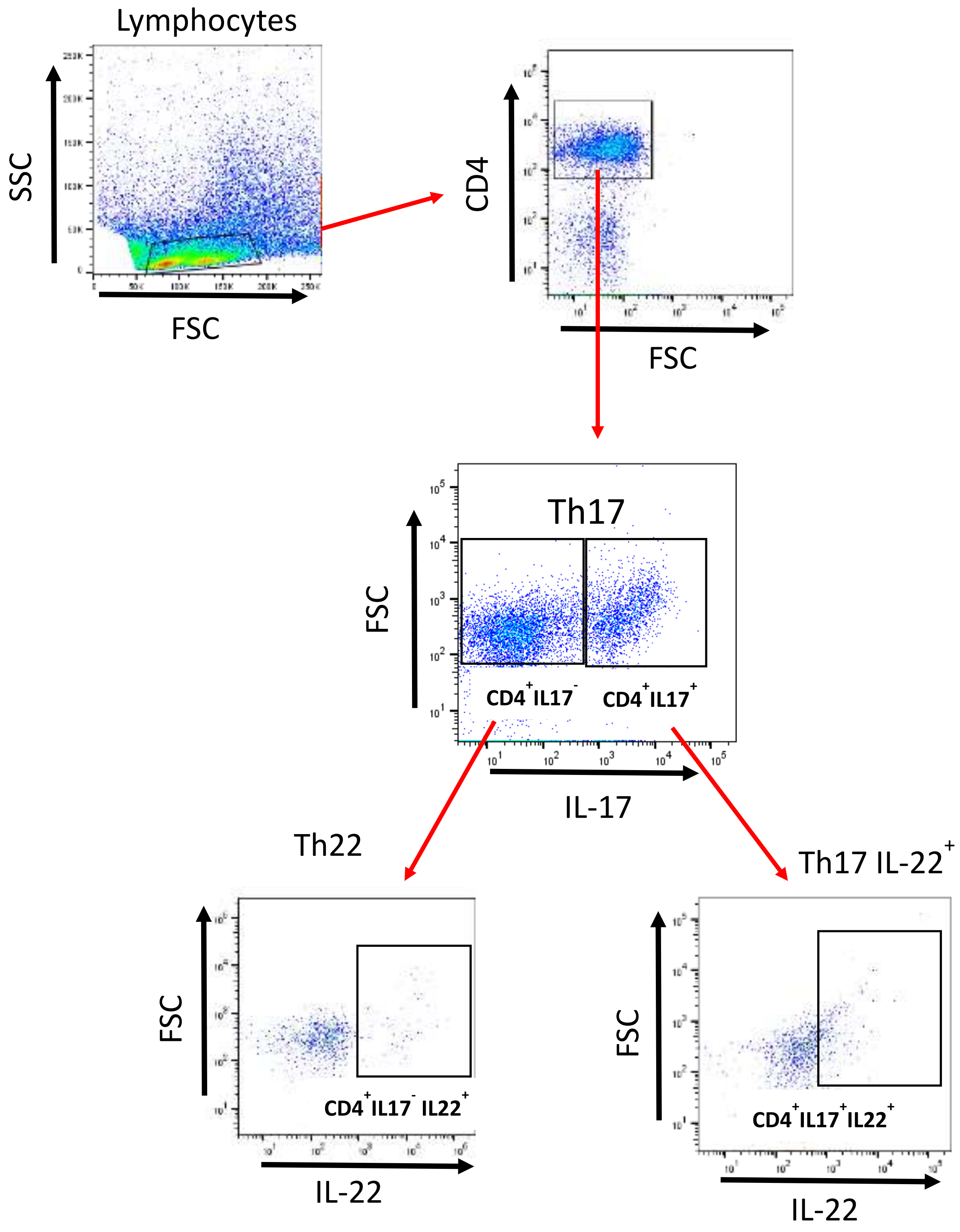

S5 Fig Gate strategy used to define Th22 and Th17 CD4⁺ T cell subsets. Lung infiltrating leukocytes were obtained at several post-infection periods, granulocytes gated by SSC and FSC scatters. Cells were then assessed for the expression of membrane CD4 and then for the presence of intracellular cytokines (IL-17 or IL-22).
